# Phenolic Compounds in *Salicornia* spp. and Their Potential Therapeutic Effects on H1N1, HBV, HCV, and HIV: A Review

**DOI:** 10.3390/molecules28145312

**Published:** 2023-07-10

**Authors:** Malthe Fredsgaard, Samba Evelyne Kabemba Kaniki, Io Antonopoulou, Tanmay Chaturvedi, Mette Hedegaard Thomsen

**Affiliations:** 1 AAU Energy, Aalborg University, 6700 Esbjerg, Denmark; 2 Biochemical Process Engineering, Division of Chemical Engineering, Department of Civil, Environmental and Natural Resources Engineering, Luleå University of Technology, SE-97187 Luleå, Sweden

**Keywords:** *Salicornia* spp., phenolic compounds, antiviral nutraceuticals, flavonoids, H1N1, HBV, HCV, HIV, enzymatic inhibition

## Abstract

Despite public health risk mitigation measures and regulation efforts by many countries, regions, and sectors, viral outbreaks remind the world of our vulnerability to biological hazards and the importance of mitigation actions. The saltwater-tolerant plants in the *Salicornia* genus belonging to the Amaranthaceae family are widely recognized and researched as producers of clinically applicable phytochemicals. The plants in the *Salicornia* genus contain flavonoids, flavonoid glycosides, and hydroxycinnamic acids, including caffeic acid, ferulic acid, chlorogenic acid, apigenin, kaempferol, quercetin, isorhamnetin, myricetin, isoquercitrin, and myricitrin, which have all been shown to support the antiviral, virucidal, and symptom-suppressing activities. Their potential pharmacological usefulness as therapeutic medicine against viral infections has been suggested in many studies, where recent studies suggest these phenolic compounds may have pharmacological potential as therapeutic medicine against viral infections. This study reviews the antiviral effects, the mechanisms of action, and the potential as antiviral agents of the aforementioned phenolic compounds found in *Salicornia* spp. against an influenza A strain (H1N1), hepatitis B and C (HBV/HCV), and human immunodeficiency virus 1 (HIV-1), as no other literature has described these effects from the *Salicornia* genus at the time of publication. This review has the potential to have a significant societal impact by proposing the development of new antiviral nutraceuticals and pharmaceuticals derived from phenolic-rich formulations found in the edible *Salicornia* spp. These formulations could be utilized as a novel strategy by which to combat viral pandemics caused by H1N1, HBV, HCV, and HIV-1. The findings of this review indicate that isoquercitrin, myricetin, and myricitrin from *Salicornia* spp. have the potential to exhibit high efficiency in inhibiting viral infections. Myricetin exhibits inhibition of H1N1 plaque formation and reverse transcriptase, as well as integrase integration and cleavage. Isoquercitrin shows excellent neuraminidase inhibition. Myricitrin inhibits HIV-1 in infected cells. Extracts of biomass in the *Salicornia* genus could contribute to the development of more effective and efficient measures against viral infections and, ultimately, improve public health.

## 1. Introduction

The increasing focus on the interplay between public health and bioactive compounds extracted from biomass guides researchers to shift to the most prominent groups of bioactive compounds, phenolic compounds [1]. A group of secondary metabolites known as phenolic compounds are characterized by the presence of at least one (-OH) groups attached to a benzene ring, and they often possess additional functional groups such as carboxyl, methyl, ethyl, and ketone groups. These compounds can form various structures such as homodimers, heterodimers, or polymers, including diferulic acid, chlorogenic acid, or condensed tannins [2]. Phenolic compounds have been found to possess different in vitro and in vivo properties; e.g., anti-inflammatory, anticarcinogenic, antidiabetic, pain relieving, and neuroprotective [3,4,5,6]. Though the concentrations and the presence of phenolic compounds have been studied and documented in various fruits [7], nuts [8], and berries [9,10], the scientific interest in phenolic compounds from halophytic biomasses is new. Utilizing the residual biomass at the end of the annual cultivation cycle of edible halophytes could introduce a novel, holistic, circular economy view of biobased nutraceuticals, as compounds found in end-of-cycle halophyte fractions are known for their pharmacological properties [11,12,13,14]. 

*Salicornia* spp. are edible plants in the Amaranthacae family and are otherwise also known as glasswort and marsh samphire, amongst others, and are known to contain a broad array of phenolic compounds [15,16,17,18,19]. Ferulic acid is one of the many phenolic compounds found in *Salicornia* spp., and other selected phenolic compounds found include caffeic acid, chlorogenic acid, apigenin, kaempferol, quercetin, isorhamnetin, myricetin, isoquercitrin, and myricitrin [15,20,21,22,23,24]. Species belonging to the *Salicornia* genus have been underutilized as medicinal herbs in recent decades, even though they have been traditionally consumed for their flavorful aerial parts in salads and seafood dishes. Additionally, they have been used as a green salt alternative, which results in a lower risk of cardiovascular diseases compared to regular salt [25,26,27,28,29,30,31,32]. The average person’s daily intake of fruit and vegetables has decreased with the introduction of processed foods containing high amounts of simple sugars, starch, sodium, and fat. As fruits, vegetables, and herbs are the primary sources of phenolic compounds, this change in diet towards heavily processed food rich in macronutrients also decreased the intake, and thereby the nutraceutical effects, of phenolic compounds, thus potentially increasing the risk of lifestyle-related diseases and severe viral infections [14,33,34,35,36]. Limongelli et al. reviewed phenolic compounds from the *Salicornia* genus and their health-promoting effects. In their review, they stated that the species in the *Salicornia* genus possess antitumor, antihypertensive, antibacterial, neuroprotective, and antidiabetic activities. However, antiviral effects were not mentioned [37]. High sodium diets have been proven to induce vascular dysfunction, and in a study by Panth et al., a diet with a similar high sodium concentration provided from extracts of *Salicornia europaea* L. did not induce vascular dysfunction in rats. This effect was thought to be due to ferulic acid, which was associated with nitric oxide regulation in a study by Suzuki et al. [26,38]. *Salicornia* spp. possess many medicinal properties attributed to the phenolic content, many of which contain multiple inhibitory pathways towards enzymes related to pathogenesis, some completely unrelated to the antiviral protein inhibitory effect [39,40]. Among the many pharmacological applications of *Salicornia* spp., these are plants of great interest as traditional medicine against hepatitis, constipation, nephropathy, and diarrhea, and are proven to possess antihyperglycemic and antihyperlipidemic activities, thus making *Salicornia* spp. effective as a potential glucose- and lipid-regulating agent to subjects fed with high-glucose and high-lipid diets [27,41,42]. Of the phenolic compounds reviewed are two known and generally recognized as safe (GRAS) by The United States Food and Drug Administration (USFDA) and can be administered in concentrations of 500 mg/serving (quercetin); 150 mg/kg and 1500 mg/kg in food and chewing gum, respectively (isoquercitrin). USFDA is an administrative entity that discusses chemical stability, manufacturing procedures, metabolism, toxicology, clinical studies, and the reason for granting GRAS status to specific substances. At the time of publication, 1040 chemical compounds, extracts, fiber residues, proteins, etc., are registered as GRAS, and 68 with a pending decision by USFDA [43]. The other phenolic compounds in this review are not yet GRAS compounds but have been used by independent researchers worldwide for in vitro and in vivo experiments in less than toxic concentrations for their beneficial nutraceutical and pharmaceutical properties and have been deemed safe in amounts obtained through dietary consumption [44,45].

Oliveira-Alves et al. [28] attributed the variations in phenolic compound concentrations observed in *Salicornia ramosissima* J. Woods from Table 1 to several factors. These factors include the maturity of the biomass, physical pretreatment methods such as milling or shredding, drying pretreatment techniques such as oven-drying or freeze-drying, and the choice of extraction methods. These factors may account for the significant differences in the measured concentrations of ferulic acid and chlorogenic acid, as presented by Oliveira-Alves et al. and Silva et al. [18,28].

Phenolic compounds are often not very soluble in water, and the scalability of conventional extraction methods using flammable organic solvents is complex, making extracting naturally derived phenolic compounds expensive. Chaturvedi et al. reviewed the extraction of phenolic compounds from lignocellulosic biomasses, hereunder *Salicornia* spp. They concluded sub-critical water extraction to be a cost-effective extraction for phenolic compounds on an industrial scale, for which the compounds have also been proven valuable for biorefinery purposes, as concluded by other authors [13,48,49]. The group of deep eutectic solvents (DES) has emerged as a promising solvent group for the extraction of phenolic compounds. Qin et al. [50] used DES to greatly increase the solubility of phenolic compounds in the solvent while still being a harmless solvent for humans and the environment. Similar effects have been seen by using an ionic liquid, closely related to DES, for the extraction of phenolic compounds from a *Salicornia europaea* matrix, recently performed by Wang and Wang, who found a significantly higher extraction efficiency using 1-butyl-3-methylimidazolium chloride salt in water than using pure water or 70% ethanol as extraction solvents [51]. As the extract obtained after any extraction of phenolics is significantly diluted, as seen in Table 1, isolation and purification are necessary to yield a pure and concentrated fraction of phenolics. Industrial-scale isolation poses a challenge, as many laboratory-scale isolations from aqueous solutions use aqueous-organic liquid-liquid extractions. However, scaling of liquid-liquid extractions can be of concern due to energy-intensive solvent recovery, flammability, and toxicity [48]. Soto et al. reviewed the flexibility and options for resin adsorption and desorption of phenolic compounds, and a common finding across all the studies reviewed was a high concentration of phenolics in the desorbate, which can be selectively desorbed from the resin by altering the desorption solvent, allowing for the separation of different classes of phenolics, hence using far less solvent than a liquid-liquid extraction [52]. 

While vaccines play a crucial role in preventing the spread of many viruses, antiviral drugs are used to treat viral infections once they have occurred. Currently, available antiviral drugs are limited in number and effectiveness, and the development of new antiviral agents is challenging due to the rapid evolution of viruses and the development of drug resistance by viruses [53,54]. Epidemic and pandemic outbursts of viruses have been shown to kill millions. The influenza A (H1N1)-induced Spanish Flu (1918–1920) has various estimations regarding the death burden, but a realistic estimate was 17.4 million [55]. Furthermore, despite the attempts of worldwide influenza vaccinations, 1 billion cases are estimated yearly, resulting in between 290,000 and 650,000 deaths [53,56]. Estimates of the amount of hepatitis virus carriers globally vary greatly due to long incubation times for the virus, with ranges from 257–290 million for hepatitis B virus (HBV) and 71–300 million for hepatitis C virus (HCV) [57,58]. Of this amount, only 15 million people worldwide have been diagnosed with HCV; 439,000–1,000,000 people were under treatment for HCV in 2015 [58,59]. An estimated annual 887,000 and 385,000 deaths were attributed to HBV and HCV infections, respectively, where the majority of the deaths could have been avoided by timely detection and treatment [60]. This review will only cover HBV and HCV infections, as these account for most infections and deaths of hepatitis infections, even though the worldwide contribution towards treating chronic HCV has more than 10-fold increased from 439,000–1,000,000 to 9,400,000 from 2015 to 2021 [58]. Infections and treatment vary as infected patients might carry the viruses in inactive states for decades, unknowingly spreading the disease to sexual partners and, for women, their newborns. A vaccine has been developed for HBV but one has yet to be developed for HCV. However, once infected by HCV, an oral treatment for 8–24 weeks is usually enough to cure the patient, but combination direct-acting drug therapy can decrease the treatment period [58,59]. Chronic symptoms usually develop after many years of infection, with the fully established virus in the patient’s system. Symptoms can vary from none to cirrhosis and hepatocellular carcinoma (HCC), and many patients are unaware of being carriers of the virus, risking infecting people that will show symptoms [57,58,59,61,62]. Antiviral resistance has been found in up to 76% of HBV patients receiving the reverse transcriptase (RT) inhibiting the nucleoside analog lamivudine for five years or more [63]. This, amongst other factors, has led to the development of more potent synthetic antiviral drugs. Such newer drugs, e.g., tenofovir and entecavir, have proven much lower antiviral resistance of <1% in 2 years of treatment in treatment-naive patients, as at least three RT substitutions induced by HBV-DNA mutation are required to develop antiviral resistance, implying the importance of continuous research in antiviral therapeutics for early-stage infected or prevention [63,64].

Phenolic compounds have been shown to possess antiviral activity against a range of viruses, which serves as an inspiration for developing new antiviral drugs. The potential antiviral properties of phenolic compounds found in *Salicornia* spp. have been the subject of recent research. Studies have shown that *Salicornia* spp. contain a range of phenolic compounds, which have been individually proven to exhibit antiviral activity through inhibition of H1N1, HBV and HCV, and human immunodeficiency virus 1 (HIV-1), see Table 1. The antiviral activity of phenolic compounds is due to their ability to interact and inhibit viral and host proteins and enzymes, preventing viral replication and infectivity [65,66,67,68]. Among the many research groups investigating the viral inhibitory effect of phenolic compounds, Loaiza-Cano et al., Xu et al., Besednova et al., Ono et al., Badshah et al., and Nagarajan researched the anti-viral properties of more than 100 selected phenolic compounds, and concluded that phenolic compounds with a high number of (-OH) groups and certain specific positions obtained the highest inhibitory effect. Many of the compounds with virus-inhibitory effects were found in plants known as traditional medicinal plants or foods [69,70,71,72,73,74]. Chen et al. reviewed the bioavailability of phenolic compounds and found that free phenolic compounds, e.g., quercetin, are presumed to partly degrade through dehydration reactions to hydroxybenzoic acids through gastric absorption, as the environment of the gastrointestinal tract possesses high bacterial conjugative enzyme activity [75]. By maintaining the phenolic compounds in a food matrix as bound phenolic compounds, many of the phenolic compounds can be delivered through the colon, hence avoiding degradation in the small intestine, and higher concentrations can be detected in the blood plasma. However, this adsorption method only delivers low concentrations of phenolic compounds in an untargeted manner [76,77]. The pharmacokinetics plasma concentration-time profiles for the adsorption of phenolic compounds from *Echinacea purpurea* extract were studied in a rat model by Gan et al., who found that adsorption of chlorogenic and caffeic acid through ingestion occurs in 15 and 360 min with associated disappearance half-times of 7.72 and 6.00 h, respectively [78]. This indicates that after the adsorption of phenolic compounds in rats, 10% of the initial adsorption concentration will be available after 25.6 h for chlorogenic acid and 20 h for caffeic acid. If this is also the case in humans, administering phenolic compounds towards a viral infection to obtain optimal response from the phenolic compounds should be precisely timed, and possibly administered multiple times daily during the infection period. Serra et al. investigated the distribution and accumulation of luteolin, a flavonoid closely related to quercetin, in a rat model. They found a quick distribution of luteolin into the kidneys, testicles, and heart, and no luteolin was detected in the liver, brain, thymus, and spleen, which might indicate a poor effect from luteolin to inhibit hepatitis infections in the liver. However, very low concentrations of luteolin were detected compared to the phenolic acids and their concentrations in the extract provided for ingestion, potentially indicating that the flavonoids degraded to other phenolic compounds [79]. If the research by Serra et al. also holds for other flavonoids, this could indicate the necessity for stabilizing flavonoids by encapsulation, nanoemulsion, or as a glucan conjugate for targeted delivery of flavonoids. 

This study reviews phenolic compounds of *Salicornia* spp. and their potential antiviral effect on H1N1, HBV, HCV, and HIV, as these viruses continue to have serious effects on human health globally and could induce pandemic outbreaks if strains develop multi-resistance towards available treatments. 

## 2. H1N1

**Figure 1:**
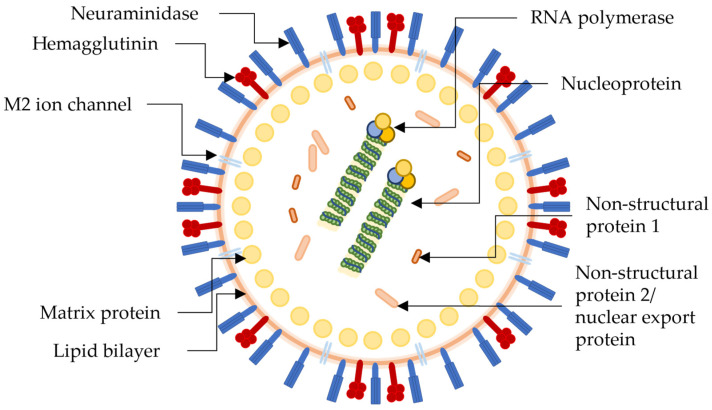
Pictorial representation of the structure and biology of a generic influenza virion with antiretroviral drug targets. Not specific to the H1N1 virus.

The influenza virus is a part of the *Orthomyxovirus* family that contains four types; influenza A to influenza D, with only influenza A to influenza C being able to infect humans, with the H1N1 strain of influenza A being the most predominant of all. H1N1 contains the active site proteins, as seen on the H1N1 virion in Figure 1, hemagglutinin (HA), neuraminidase (NA), and M2 ion channels, which antiviral therapeutics can inhibit. Multiple subtypes of influenza have been described, including 18 different subtypes of HA and 11 subtypes of NA, classified in 162 influenza A subtypes, with a theoretical amount of 198 being possible [56,80]. NA and HA are surface glycoprotein spikes, usually in a ratio of 4 NA to 1 HA. 

**Figure 2:**
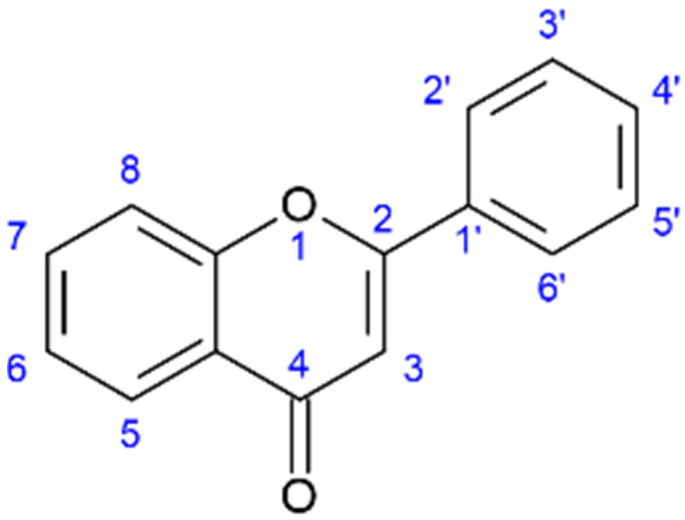
General structure of the flavone backbone (2-phenyl-1,4-benzopyrone) and numbered sites for attaching, amongst others, hydroxyl, methyl, and sugar groups.

Liu et al. concluded that the inhibitory effects of flavonoids towards NA were highly structure-dependent and established a general order of potency: aurones > flavonoles > isoflavones > flavanonoles and flavanoles. For the optimal inhibitory effect of NA, specific hydroxyl group placements were established at positions 4′-OH and 7-OH, the carbonyl group on C4=O, and a set of double-bound carbon C2=C3; see Figure 2 for reference. Compounds from *Salicornia* spp. in Table 1 that have these properties are kaempferol, apigenin, myricetin, quercetin, myricitrin, and isoquercitrin. The most potent NA inhibitory effects from the study of 25 flavonoids by Liu et al. were from the flavones apigenin, luteolin, not having the 3-OH hydroxyl group, and the aurones flavonoid group [65].

Isoquercitrin is the 3-*O*-glucoside form of quercetin, making the compound more polar than the aglycone and with higher bioavailability [40]. Even though the structural differences between glycosylated flavonoids can be minor, a clear difference between the closely related isomers isoquercitrin and hyperoside could be observed between the antiviral effects of the compounds when a green fluorescent protein (GFP) reporter was inserted into H1N1, and the modified virus was mixed with the flavonoids at 25 µM and compared to a negative assay control (mock). An insignificant effect was seen between the effect from the mock and hyperoside, but isoquercitrin resulted in almost complete inhibition of H1N1-infected chicken embryos. The H1N1 inhibiting effect provided by isoquercitrin was likely the primary reason for the H1N1 inhibiting effect seen by a water extract of *Nelumbo nucifera* Gaertn. [81]. As isoquercitrin has been found in great concentrations in *S. fruticosa* L., see Table 1, a purified fraction of isoquercitrin from the *Salicornia* species could also possess high inhibition of H1N1 in chicken embryos. Isoquercitrin has been shown to exhibit the suppression of hemagglutinin (HA) and NA and inhibit cytopathic effects on both H1N1 and H3N2, making isoquercitrin a multi-acting therapeutic agent [82]. The anti-H1N1 effect of isoquercitrin has also been linked to the suppression of reactive oxygen species (ROS), acidic vesicular organelle formation, and the polymerase basic protein 2 (PB2) protein [83]. Kim et al. observed a significant synergistic effect when isoquercitrin was combined with amantadine in low concentrations and did not induce resistant vira. Isoquercitrin showed a higher inhibition rate of the H1N1 virus than (-)-epigallocatechin gallate and resveratrol, which have previously been known to have a high inhibition rate of the H1N1 virus when compared to other antiviral phenolic compounds [54]. 

During the past few years, ferulic acid has been determined to play an essential role as a nutraceutical and against viral infections, including H1N1 with an IC_50_ inhibition at 140 µM towards NA [84]. In addition to low cytotoxicity, ferulic acid and caffeic acid show utility for regulating the activity of the immune system [85,86]. Sodium ferulate activated toll-like receptors (TLR) 7 and 9, enhancing survival in H1N1-infected mice, contributing to antiviral defense [87]. This effect could be secondary to the induction of the heme oxygenase 1 gene (HMOX1) and possibly reflect ferulic acid’s additional benefits. TLR7 and TLR9 are proteins responsible for detecting viral nucleic acids, initiating swift signaling pathways that play a crucial role in generating interferons, thereby enhancing the body’s antiviral defenses [87]. Another potential impact of ferulic acid is its inhibiting effect on functions such as virus replication. During the past few years, ferulic acid has been determined to play an essential role against viral infections, including H1N1, murine coronavirus (MHV-A59), and human rhinovirus 14 (HRV-14), as a component of phenolic extract from plants with exceptional and identified action [84,88,89,90,91,92,93,94]. Ferulic acid is also a cytochrome P450 1A2 enzyme (CYP1A2) inhibitor, positively increasing its half-life and averting serious drug interactions [95]. CYP1A2 is involved in the metabolism of xenobiotics in the body involved in drug metabolism. Feruloylated oligosaccharides (FOs) are nutraceuticals derived from arabinoxylans, with ferulic acid esterified to l-arabinofuranosyl side chain oligosaccharides. FOs possess the physiological functions of both ferulic acid and oligosaccharides. Unlike pure oligosaccharides, FOs still release ferulic acid after fermentation by human gut microorganisms, which can introduce prebiotic effects in the gut microbiota [96]. Ferulic acid released in the colon can be absorbed in the body, exerting various physiological functions as free ferulic acid. The anti-inflammatory impact of antioxidant nutraceuticals, such as phenolics from *Salicornia* spp., might also quell the excessive inflammatory reaction within lung parenchyma evoked by viral H1N1 [93,97]. McCarty et al. suggested that a very low daily dosage ranging from 500 to 1000 mg of ferulic acid could aid in the control of H1N1 by decreasing infection response by suppressing viral spread and dampening proinflammatory signaling, which promotes an influx of inflammatory cells [98]. 

Chlorogenic acids are a group of esters formed between caffeic and quinic acids and contain chlorogenic acid (ChA), neochlorogenic acid (neo-ChA), and cryptochlorogenic acid (crypto-ChA), amongst others. These three compounds are closely related isomers, only differentiated by the orientation of the quinic acid group. ChAs have been recognized as an effective NA inhibitor by Ding et al., who proposed it as a nutraceutical that can decrease the time of the infection and be safely administered through the diet in low concentrations; e.g., by ingesting wild lowbush blueberries [99]. Ding et al. proved that the synthetic medicine oseltamivir at 2 µM was less inhibiting on H1N1 than 100 µM ChA, and the cellular toxicity of ChA was 0% at levels below 200 µM [99]. 

Dayem et al. investigated the in vivo pharmaceutical effect of five flavonoids and their effect on H1N1-infected mice. Trials were set up with the administration of flavonoids as pre-treatment before virus inoculation, co-treatment with simultaneous viral inoculation and flavonoid administration, and post-treatment flavonoid administration after virus inoculation [100]. It was found that isorhamnetin suppressed the covalent binding of lipids to peptides and the lipidation of microtubule-associated protein 1 light chain 3 beta (MAP1LC3B), hence decreasing virus-induced overexpression of autophagy-related genes of healthy cells (autophagy-related gene proteins 5 and 7 (atg-5 and atg-7)) protecting the infected host. Isorhamnetin was proven to inhibit both NA and HA at a concentration of 50 µM, indicating the potential use of isorhamnetin as an anti-H1N1 medication [100].

As seen in Table 2, many of the phenolic compounds selected for the review have been tested for various inhibition mechanisms in silico and in vitro. Typical for many of the selected phenolic compounds is the ability to inhibit the strain A/PR/8/34 (H1N1) in an infected MDCK cell line, and the NAI of that strain has also been investigated in multiple studies. From this, the inhibition efficiency of the compounds, tested on the MDCK cell line with A/PR/8/34 (H1N1), was shown to be isoquercitrin > caffeic acid > quercetin, and the NAI of the selected compounds on A/PR/8/34 (H1N1) was shown to be apigenin > kaempferol > quercetin > myricetin. Molecular docking simulations also proved the effect on NAI with similar results to the in vitro NAI results, showing the most potent inhibitors to be isoquercitrin > ferulic acid > kaempferol > quercetin. Furthermore, ChA is seen to possess good NAI and could, together with isoquercitrin, be viable options for NAI. 

## 3. Hepatitis

Many phenolic compounds have been found to possess direct hepatoprotective properties and inhibit HBV and HCV reproduction; e.g., ellagic acid, isorhamnetin, quercetin, and myricitrin [66,67,107,108]. These hepatoprotective properties might not eliminate the root cause of the problem, the viral infection, but intentional administration of such compounds in elevated concentrations in the diet might offset, delay, or reduce the symptoms related to HBV in a non-vaccinated individual by acting as an agonist, antagonist, or synergistic by inducing or inhibiting protein and enzymatic multi-pathways [107,109]. On the other hand, quercetin has been found to act as an antagonist by inhibiting the multi-enzymatic activity non-structural protein 3 (NS3) protease, NS5B, and envelope glycoprotein 2 protein, hence stopping HCV replication. The flavones closely related to quercetin, luteolin and apigenin, showed similar binding energy through in silico molecular docking results of NS5B compared to the synthetic NS5B-inhibiting drug sofosbuvir [67,68,110]. Although sofosbuvir is a second-generation direct-acting antiviral drug with fewer side effects, it is still known to induce a series of side effects, e.g., headaches, fatigue, arthralgias, diarrhea, nausea, and anemia, in patients for whom it is administered. A drug substitution using a naturally occurring GRAS NS5B-inhibiting nutraceutical, such as quercetin, could eliminate the drug-related side effects and generally increase the wellbeing of the patient, as the majority of the compounds are not only direct-acting but are, in general, beneficial for the entire body [111].

**Figure 3:**
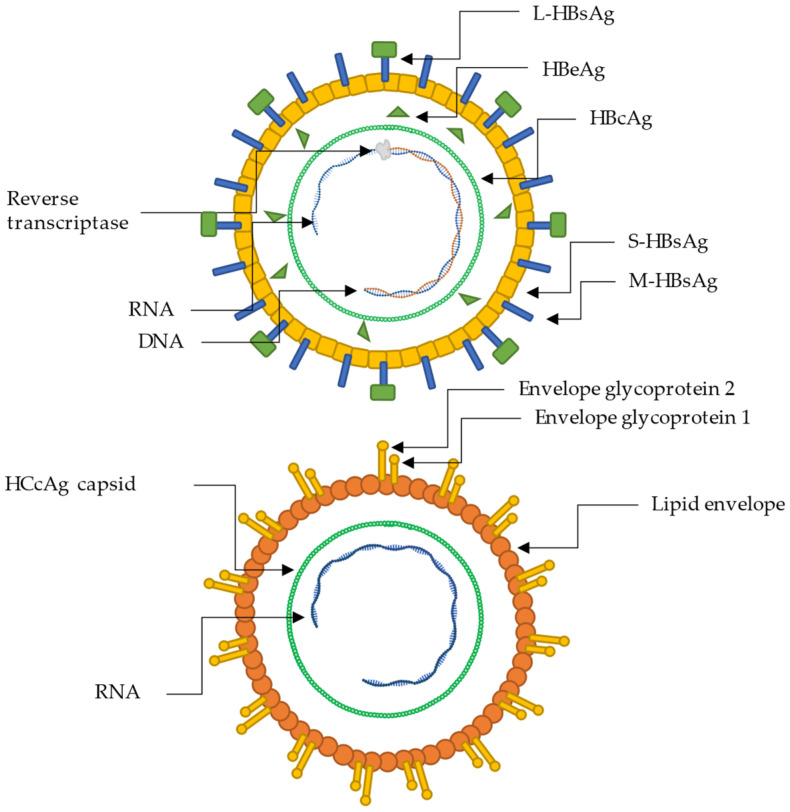
Pictorial representation of the structure and biology of hepatitis B and C virions. The upper virion represents hepatitis B, and the lower virion represents hepatitis C.

Quercetin and myricitrin have been found to inhibit the HBV surface and e antigens, HBsAg and HBeAg; see Figure 3 [16,66]. Quercetin and myricitrin were isolated from *Guiera senegalensis* J.F. Gmel leaves by 96% ethanolic 72-h maceration, followed by rotary evaporation of the solvent, and were sequentially partitioned using hexane, chloroform, and n-butanol. The lipophobic n-butanol fraction was fractionated using normal phase column chromatography into eight sub-fractions. Secondary column chromatography was performed, and the pure compounds were obtained and verified using 2D NMR spectral analysis by ^1^H-NMR and ^13^C-NMR. The isolated compounds, quercetin and myricitrin, were analyzed in silico by molecular docking, and both compounds showed good docking scores towards all analyzed proteins, RT, Na^+^/Taurocholate Cotransporting Polypeptide (NTCP), and HBcAg. NTCP, a protein membrane transporter in healthy liver cells, has also been proven to be a receptor for HBV entry, and therapeutics can act as host receptor restrictors and block HBV entry [112]. Quercetin and myricitrin were tested by a human hepatoblastoma cell line cytotoxicity assay and determined to be safe in the liver up to 165 µM and 107 µM, respectively, and these concentrations were therefore used in the HBsAg and HBeAg enzyme bioassays. Quercetin exhibited excellent HBsAg inhibition and HBeAg downregulation of 60% and 62%, respectively, compared to myricitrin by 44% and 35%, respectively. The effect of quercetin was close to that of the HBeAg-targeting synthetic lamivudine (69% at 2 µM) [66]. The aglycone of myricitrin, myricetin, also shows anti-hepatitis and hepatoprotective properties in acute hepatitis and more severe fulminant hepatitis. Myricetin was shown to exert anti-programmed cell death as antiapoptosis of healthy liver cells, together with the antioxidant and anti-inflammatory effects, further potentially decreasing the risk of symptoms. The antiapoptosis effect was explained by the regulation of the cell metabolism enzymes cysteine-dependent aspartate-directed proteases 3 (caspase-3), caspase-9, protein p53, and the inhibition of nuclear factor-kappa B (NF-κB) activation [113]. Caspase-3 and caspase-9 are responsible for executing and initiating apoptosis, respectively, for healthy cells, and regulation hereof is essential for killing infected cells and avoiding or postponing severe hepatitis symptoms, hence ensuring natural cell metabolism [114]. During an HBV infection, the protein responsible for caspase-3 regulation is the virus-encoded hepatitis B virus protein X (HBx), produced by the host cell upon infection as a gene product [115]. HBx is, together with other proteins, responsible for activating the transcription of viral genes and HCC, and can induce or block regular metabolism apoptosis, and was shown to do so through caspase-3 inhibition [116,117]. HCC is a common type of primary liver cancer that can, amongst other things, be caused by the down-expression or inactivation of phosphatase and tensin homolog (PTEN), a tumor suppressor gene. HCC caused by HBV is one of the most common cancer variants, and the mortality rate is projected to increase by 40% from 2019 to 2030 [118,119]. Multiple phenolic compounds, e.g., gossypetin and naringenin, have been shown to attenuate the metabolic changes directly caused by the HBx protein [120]. This decreases apoptosis, hepatitis-related hepatic steatosis, HCC, and cirrhosis. The antiapoptosis effect, as seen by myricetin, might be expected to be similar, as some of the compounds responsible for the inhibition are close isomers, even though it has not yet been described [107,120].

Zhang et al. investigated the inhibitory effect of phenolic compounds on proteins and enzymes involved in HBV replication, inflammatory response, and interaction with other proteins and enzymes [121]. Zhang et al. introduced a target network of 13 potential targets for treating HBV, and among these were the signal transducer and activator of transcription 3 (STAT3) and euchromatic histone-lysine N-methyltransferase 2 (EHMT2). These two proteins are responsible for taking place in the virus replication of HBV and down-regulating the host’s immune response to the virus. ChA was seen, through molecular docking simulations, to partly inhibit both STAT3 and EHMT2, whereas caffeic acid partly inhibited EHMT2, ensuring optimal host immune defense upon infection [121].

With hepatitis viruses being necroinflammatory and hepatotropic, the anti-inflammatory effect of nutraceuticals on the liver’s vital functions in the late stage of the infection can be highly beneficial to attenuate the virus-induced inflammatory response [122,123]. Proinflammatory enzymes and cytokines, such as interleukin-1 beta (IL-1β), inducible nitric oxide synthase (iNOS), and cyclooxygenase-2 (COX-2), are produced by HCV gene expression upon infection, and the suppression of these specific enzymes and cytokines are found from phenolic compounds found in green tea, many of which are also present in the plants of the *Salicornia* genus [123]. Ma et al. investigated the protective effect of quercetin against CCl_4_-induced inflammation by significantly decreasing the production of IL-1β, iNOS, and COX-2 [124]. The anti-inflammatory response from quercetin by Ma et al. was achieved by inhibiting TLR2, TLR4, and NF-κB. TLR2 and TLR4 have both been found during infection by HCV and HCV/HIV-1 co-infection, indicating that the inhibitory effect of quercetin towards TLR2 and TLR4 could also apply during viral infections, lowering the inflammatory response of the patient [124,125].

Both HBV and HCV can trigger ROS production in the mitochondria of the infected hepatocytes of the host cells, hence causing mitochondrial dysfunction. Neoplastic cell transformation of the said host cell can cause oxidative stress by increased ROS levels, possibly due to the interference with the host cell’s DNA reparation systems for the removal of oxidized DNA, increasing the risk of HCC. ROS also promotes proinflammatory cytokine production, increasing the liver’s inflammatory response [126]. When infected with HBV, the intracellular produced HBx will identify the mitochondria and increase ROS levels towards 10,000-fold of normal levels, which can cause 20 types of DNA mutations [127,128,129]. Administering phenolics found in *Salicornia* spp., natural antioxidants, and hence ROS scavengers, to HBV and HCV patients, intracellular hepatic ROS production can be limited and delay HBV- and HCV-related liver carcinogenesis, HCC, and hepatic fibrogenesis [130,131]. 

As seen in Table 3, the literature available shows sparse data regarding the interactions between HBV or HCV and the phenolic compounds selected for this review from *Salicornia* spp. Some molecular docking studies, which tend to be the first indication of potentially new drug candidates, have been performed on the proteins and enzymes responsible for viral entry and replication and the phenolic compounds of interest. From this data, the most promising compounds for HBcAg inhibition are kaempferol > quercetin > myricitrin. The most promising HBV-RT inhibition compounds are kaempferol > quercetin > myricitrin. In addition to these inhibitions, ChA shows good HBV-DNA inhibitory effects at low concentrations compared to caffeic acid, but poorer inhibition of HBsAg and HBeAg, two crucial targets for anti-HBV therapy. 

## 4. HIV

**Figure 4:**
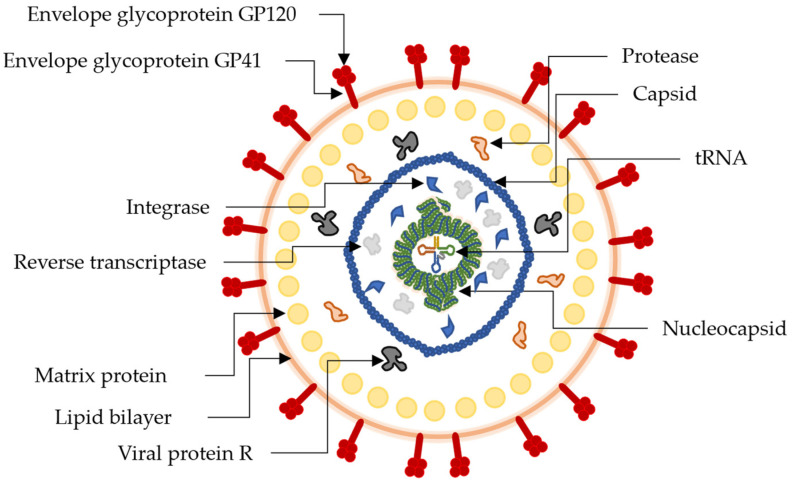
Pictorial representation of the structure and biology of an HIV-1 virion with antiretroviral drug targets.

HIV-1 is a lentivirus the virion of which is a highly complex structure enveloped by a lipid bilayer with two glycoproteins attached (GP41 and GP120) and contains a multitude of proteins including protease (PR), integrase (IN) and RT, see Figure 4, all being actively targeted for anti-HIV-1 mechanisms by therapeutic antagonists. During virus replication and infection, a polyprotein consisting of RT, PR, and IN is split by the PR. The single-stranded RNA genome of the virus then relies on the RT and IN to catalyze the viral DNA integration into the cluster of differentiation 4 (CD4) host cells. After integration, GP41 and GP120 progressively mature, resulting in a stronger viral envelope over time. As GP120 and GP41 are glycoproteins, they have been proven to be inhibited by glucosidase through cleavage [135]. HIV-1 antiretroviral therapy often targets RT, PR, or glycoproteins using pharmacophore compatibility, such as protein-ligand docking simulations [135,136]. Polyhydroxylated compounds such as caffeic acid, myricitrin, quercetin, or kaempferol have been reported as natural pharmacophores for HIV-1 [69,89,136]. IN’s 3′-P central core domain contains a metal-binding active site with three amino acid residues specific for Mn and Mg cations. During infection, the IN-DNA complex is formed and later stabilized by IN. The high affinity of viral polyproteins against polyhydroxylated phenolic compounds reveals their ability to compete with various co-factors and form protein-ligand complexes [136,137]. 

A study by Bailly et al. found that caffeic acid interacts with the viral envelope GP120, which is essential for the virus to bind and enter the host cell [138]. GP120 binds and fuses with the T lymphocytes using its variable regions V2, V3, and V4. These glycoprotein regions form loops to neutralize the antibodies and are crucial in the virus-host interaction [139]. Repeated passages of the virus in the presence of caffeic acid resulted in the inhibition of the V2-V4 regions of GP120. Caffeic acid-induced inhibition can alter the virus’s ability to bind and penetrate past the host cell receptors [138]. The HIV-1 IN has the ability to perform the G140S mutation, in which glycine (G) is replaced with serine (S) at 140 base pair. This effect was found to be inhibited by caffeic acid, and hence the stops the mutation from happening. The mutation gives antiviral resistance against pharmaceutical IN inhibitors, creating a negative feedback loop for the infected patient [140,141]. However, the catechol group of caffeic acid makes it an efficient cleavage inhibitor for IN despite the mutation [137,142]. The results suggest that caffeic acid and its derivatives could be promising candidates for developing anti-HIV-1 therapeutic agents.

In a study by Yang et al., leukaemia cells, C11 Jurkat, were infected with the HIV-1 provirus flanked and controlled by the long terminal repeat (LTR) regions [143]. The aim was to quantify the provirus transcription with a GFP as a selection marker. After 72 h of constant exposure to quercetin at 20 µM, the HIV-1 transcription in latently infected cells was increased to 14.5%. A positive correlation between the concentration of quercetin and the HIV-1 expression was observed. The expression of the provirus was observed by the presence of fluorescence from the GFP in fluorescence microscopy. GFP-positive cells increased over time to 14.6% until 72 h and with increased concentration of quercetin until 20 µM [144]. In the attempt to understand which transcription factor activates the provirus expression, the transcription factor NF-κB was silenced before inducing transcription. The induction of HIV-1 LTR (Δ*κ*B) with the tumor necrosis factor-alpha (TNF-α) failed to show any HIV-1 expression. This finding indicates that quercetin uses the NF-*κ*B signaling pathway to activate the viral transcription. Quercetin’s ability to activate the expression of retroviral genes in latent cells is extremely useful in the development of therapy against HIV-1, as latency of HIV-1-infected cells continue to be a risk for affected patients. Therefore, it is valuable to reactivate the dormant cells with a safe natural agent such as quercetin. It could make an effective supplement for co-treatment with a High Active Antiviral Therapy (HAART) drug in order to reactivate the latent infected cells and introduce the HAART drug, in a ‘shock-and-kill’ strategy [144]. Neither closely related isomers of quercetin or quercetin derivatives saw high inhibition of HIV-1, and if the 3′ hydroxyl group was placed on the 2′ position, yielding the isomer morin, the inhibitory effect was not present [69]. On the other hand, adding a hydroxyl group on the 5′ position, yielding myricetin, an increased inhibitory effect against RT was observed [69,135]. The mechanism of this flavonoid relies on its ability to compete with the template primer of RT [145]. Myricetin was found by Fesen et al. to possess high IN cleavage efficiency at 67.8%. Not only was myricetin found to be a good RT inhibitor, but also a good inhibitor of certain ATP-dependent enzymes, such as the plasma membrane Ca^2+^ pump, and could therefore increase the potential for pharmacological applications [146]. Engineered TZM-bl cells sampled from the uterine epithelial tissue were genetically manipulated to express the CD4 co-receptor, were exposed to myricetin and showed >87% inhibition of HIV-1 at a concentration of 100 µM. Those findings were supported in another study by Ortega et al., where myricetin showed HIV-1 inhibition of >80% with an IC_50_ of 7.6 µM [147]. This proves isolated myricetin from *Salicornia* spp. to be a compatible flavonoid for developing anti-HIV-1 topical microbicides [147]. 

Apigenin showed inhibition of HIV-1 expression in contaminated H9 cells from the T cell line [147,148]. Similar to quercetin, apigenin interacts with the transcription factor NF-κB at a concentration as low as 10 µM [148]. This is crucial to regulate viral transcription and replication by binding to the HIV-1 promoter region in the host cell’s DNA. Apigenin competes with NF-κB to stop the viral transcription while keeping relatively low toxicity. 

As seen from Table 4, isorhamnetin, myricetin, and quercetin show promising preliminary results and should be further investigated for other anti-HIV-1 properties in clinical trials through extraction and isolation from *Salicornia* spp. to investigate their safety, efficiency, and potential widespread use in treating HIV-1. These three phenolic compounds, administered as single compounds or in a mix for potential synergistic effects, could have the potential to achieve optimal inhibitory effects against HIV-1 by targeting IN, RT, TNF-α, and NF-κB. The synergistic interaction of these compounds, due to high inhibitory activities on various target mechanisms, could lead to greater inhibition of the virus than using a single compound alone. Despite the various in vitro cytotoxicity and inhibition analyses of the single compounds, further research and clinical trials are needed to fully evaluate the safety and inhibition efficiency of the combination of flavonoids from *Salicornia* spp., but it holds promise as a potential treatment for HIV-1 infections. 

A large-scale study on the molecular docking energies of all the selected compounds should be conducted on IN, RT, capsid, PR, matrix protein, GP120, GP41, etc., to better understand the HIV-1 protein and enzyme inhibitions. 

## 5. Discussion

To ensure correct dosage and avoid possible toxicity, the hormesis relationships between the dose of a purified *Salicornia* spp. nutraceutical extract and its effect on the targeted virus needs to be established. Some of the hormesis relationships are between the host membrane protein NTCP, other viral host receptors, and the administration of phenolic compounds found in *Salicornia* spp. Optimal function of NTCP is necessary to maintain optimal liver function, so overinhibition of this host membrane protein to exclude viral infection can be damaging for liver cells [66,112,157]. Multiple hormesis effects have also been seen from increased phenolic intakes towards cancer therapy, as excessive intake has been shown to increase the risk of acute myeloid leukemia in infants [158,159]. However, phenolic compounds have been found to exert antitumor functions by inhibiting leukemic cell growth, and in a recent study, quercetin was found to be applicable in leukemic cancer therapy [160]. Hormesis effects from the pure fractions of *Salicornia* spp. should be investigated to avoid an increased risk of adverse effects as an extraordinarily high concentration of flavonoids, e.g., myricetin, have been found [18,44,161]. If extracts of *Salicornia* spp. are produced and fractionated, these possible adverse effects might be avoided if individual phenolic compounds, or other compounds being co-extracted and co-isolated, are discarded in the fractionation process. Greater understanding of phenolic compound-disease and phenolic compound-symptom hormesis relationships should be obtained before using *Salicornia* spp. phenolic compound-rich extracts for administration against the diseases and symptoms discussed in this review [44].

The reviewed phenolic compounds with the highest concentrations, see Table 1, are myricetin (*S. ramosissima* J. Woods, 465.5 ± 23.3 mg/kgDM), isoquercitrin (*S. fruticosa* L., 531.4 ± 9.4 mg/kgDM), and myricitrin (*S. fruticosa* L., 3314.5 ± 94.8 mg/kgDM), and a potential anti-viral therapy agent containing these compounds could be administered against H1N1, HBV, and HIV-1 infections. Inhibition of H1N1 plaque formation, IN integration and cleavage, and RT are seen from myricetin, NA from isoquercitrin, and HIV-1-infected cells from myricitrin. However, myricitrin has not been well documented for anti-viral effects and has not been well quantified in *Salicornia* spp., so its presence as an antiviral source remains largely undiscovered, unlike the array of other bioactive properties expelled by myricitrin [162,163,164,165,166]. Furthermore, as non-stabilized flavonoid glucosides release glucosides during digestion, the hydrolysis of the ether bond in isoquercitrin, yielding quercetin, will be able to exhibit a great inhibitory effect against HBV-RT and HBcAg due to the release of quercetin [167]. 

Plant extracts of *Salicornia* spp. are complex mixtures of high and low molecular compounds, and the in vitro analysis for H1N1, HBV, HCV, and HIV-1 are made using different assays by different providers, and with or without different cell lines. It is important to note that individual assays indicate specific antiviral enzymatic inhibition, and are only comparable for the specific virus and with a specific cell line, in specific comparable concentrations of the anti-viral compounds. To better understand the anti-viral therapeutical properties of the compounds isolated from *Salicornia* spp., testing each compound against a standard set of assays in vitro and using in vivo animal trials is suggested. It is also suggested that the combination of various molecule classes, including terpenoids, lectins, and proteins, are involved in the general process of anti-viral activity, such as virus adsorption on host cells or contaminated cell molecule release, as interactions of complex mixtures might give surprising synergistic effects [135]. As the phenolic compounds of *Salicornia* spp. here have been reviewed for their anti-viral effects, these findings can be used for the formulation of natural anti-viral therapeutic agents, as some phenolic compounds generally show similar inhibition activities towards the viral therapeutic targets as the known synthetic agents [82]. To ensure safe handling and processing, the optimized extraction of the selected compounds should be investigated, and a fractionated extract should be stabilized; i.e., through encapsulation with food-grade materials. Phenolic compounds have been proven to have a high destruction rate due to environmental stresses, and the encapsulation of extract is, therefore, essential for stabilization as the compounds, due to high enzymatic activity, are cleaved into smaller, less bioactive compounds [168]. 

In conclusion, these findings highlight the potential of mono- and polyhydroxylated phenolic compounds in the species of the genus *Salicornia* as a source of anti-H1N1, anti-HBV, anti-HCV, and anti-HIV-1 agents and warrant further investigation into their potential use in clinical settings.

## 6. Methodology

The review was performed through a literature search on phenolic compounds and H1N1, HBV, HCV, and HIV. The search was performed using the Google Scholar search engine from January 2023 to May 2023. The following keywords were used for targeted searches between viruses and specific phenolic compounds: H1N1, HBV, HCV, HIV, inhibition, virus, M2, reverse transcriptase, integrase, NNRT, protease, neuraminidase, hemagglutinin, envelope glycoprotein, HBsAg, HBeAg, HBcAg, HCcAg, bioactivity, caffeic acid, ferulic acid, chlorogenic acid, apigenin, myricetin, quercetin, kaempferol, isorhamnetin, myricitrin, and isoquercitrin.

## Figures and Tables

**Table 1:** Selected phenolic compounds detected in *Salicornia* spp. reviewed for their antiviral properties. Data are presented as mean values and standard deviations measured by triplicates or more samples.

Name	Synonym	Biomass Origin	Concentration mg/kg DM	Ref.
Caffeic acid	3,4-Dihydroxycinnamic acid	*S. europaea* L.	30.4	[17]
		*S. europaea* L.	28.6	[46]
		*S. fruticosa* L.	31.0 ± 0.8	[16]
		*S. ramosissima* J. Woods	14.4 ± 0.7	[18]
Ferulic acid	3-Methoxy-4-hydroxycinnamic acid	*S. europaea* L.	58.2	[17]
		*S. fruticosa* L.	67.9 ± 1.9	[16]
		*S. ramosissima* J. Woods	28.5 ± 1.4	[18]
		*S. ramosissima* J. Woods	35.7 ± 3.4	[28]
Chlorogenic acid	3-(3,4-Dihydroxycinnamoyl)quinic acid	*S. europaea* L.	45.1	[17]
		*S. europaea* L.	391.7	[46]
		*S. europaea* L.	840.0	[37]
		*S. fruticosa* L.	85.2 ± 1.5	[16]
		*S. ramosissima* J. Woods	16.7 ± 0.8	[18]
		*S. ramosissima* J. Woods	450.8 ± 115.2	[28]
Apigenin	4′,5,7-Trihydroxyflavone	*S. fruticosa* L.	<QL	[16]
		*S. ramosissima* J. Woods	4.5 ± 0.2	[18]
Kaempferol	3,4′,5,7-Tetrahydroxyflavone	*S. fruticosa* L.	<QL	[16]
		*S. ramosissima* J. Woods	4.5 ± 0.2	[18]
Quercetin	3,3′,4′,5,7-Pentahydroxyflavone	*S. europaea* L.	8.0	[17]
		*S. europaea* L.	14.8	[47]
		*S. fruticosa* L.	15.6 ± 0.3	[16]
		*S. ramosissima* J. Woods	34.0 ± 1.7	[18]
Isorhamnetin	3,4′,5,7-Tetrahydroxy-3′-methoxyflavone	*S. europaea* L.	58.9	[17]
Myricetin	3,3′,4′,5,5′,7-Hexahydroxyflavone	*S. fruticosa* L.	137.0 ± 1.0	[16]
		*S. ramosissima* J. Woods	465.5 ± 23.3	[18]
Isoquercitrin	Quercetin 3-O-glucoside	*S. europaea* L.	10.9	[17]
		*S. fruticosa* L.	531.4 ± 9.4	[16]
		*S. ramosissima* J. Woods	453.7 ± 68.8	[28]
Myricitrin	Myricetin 3-O-rhamnoside	*S. fruticosa* L.	3314.5 ± 94.8	[16]

DM: Dry matter. <QL: Below quantification level.

**Table 2:** Selected phenolic compounds detected in *Salicornia* spp. reviewed for their H1N1 inhibitory properties. Data are presented as mean values and standard deviations measured by triplicates.

Compound	H1N1 Inhibition Target	Inhibition Activity	Ref.
Caffeic acid	A/PR/8/34 (H1N1) infected MDCK	IC_50_ inhibition: 81.6 ± 8.3 µM	[101]
	A/Osaka/2024/09 (H1N1) infected MDCK	IC_50_ inhibition: 98.8 µM	[101]
	A/Osaka/71/11 (H1N1) infected MDCK	IC_50_ inhibition: 97.1 µM	[101]
Ferulic acid	NA	Docking energy: −7.1 kcal/mol	[84]
	A/Malaysia/Muar/33/09 NA (H1N1) inhibition (NAI) in vitro assay	IC_50_ inhibition: 140 µM	[84]
	EC_50_ of A/Malaysia/Muar/33/09 (H1N1) in infected MDCK cells	EC_50_ inhibition: 1.32 ± 0.08 µM	[84]
Chlorogenic acid	EC_50_ of A/PR/8/34 (H1N1) in infected MDCK cells	EC_50_ inhibition: 44.87 µM	[99]
	NAI by fluorescence-based in vitro assay	IC_50_ inhibition: 22.13 ± 1.07 µM	[99]
Apigenin	EC_50_ of A/PR/8/34 (H1N1) in infected MDCK cells	EC_50_ inhibition: 56.7 ± 11.1 µM	[102]
	EC_50_ of A/Toyama/129/11 (H1N1) in infected MDCK cells	EC_50_ inhibition: 65.9 ± 32.2 µM	[102]
	EC_50_ of A/Toyama/26/11 (H1N1) in infected MDCK cells	EC_50_ inhibition: 30.0 ± 17.4 µM	[102]
	A/PR/8/34 (H1N1) NAI in vitro assay	IC_50_ inhibition: 31.6 ± 0.9 µM	[65]
Kaempferol	NA	Docking energy: −6.8 kcal/mol	[103]
	NAI by RT-PCR in vitro assay	Significant inhibition at 50 µM	[100]
	A/PR/8/34 (H1N1) NAI in vitro assay	IC_50_ inhibition: 58.6 ± 0.6 µM	[65]
Quercetin	A/PR/8/34 (H1N1) infected MDCK	IC_50_ inhibition: 274.6 ± 3.3 µM	[101]
	A/PR/8/34 (H1N1) plaque formation inhibition in vitro assay	IC_50_ inhibition: 1.40 µM	[104]
	NAI by RT-PCR in vitro assay	Significant inhibition at 50 µM	[100]
	NA	Docking energy: −6.8 kcal/mol	[103]
	A/PR/8/34 (H1N1) NAI in vitro assay	IC_50_ inhibition: 58.4 ± 3.8 µM	[65]
Isorhamnetin	HI by RT-PCR in vitro assay	Significant inhibition at 50 µM	[100]
	NAI by RT-PCR in vitro assay	Significant inhibition at 50 µM	[100]
Myricetin	A/PR/8/34 (H1N1) NAI in vitro assay	IC_50_ inhibition: 82.6 ± 8.9 µM	[65]
	A/PR/8/34 (H1N1) plaque formation inhibition in vitro assay	IC_50_ inhibition: 0.90 µM	[104]
Isoquercitrin	HI RAW 264.7 cell line in vitro assay	Complete inhibition: 5 µM	[82]
	NAI by fluorescence-based in vitro assay	IC_50_ inhibition: 37.1 ± 0.6 µM	[82]
	PB2	Docking energy: −8.0 kcal/mol	[83]
	NA	Docking energy: −8.6 kcal/mol	[105]
	A/PR/8/34 (H1N1) infected MDCK	IC_50_ inhibition: 10.6 ± 0.4 µM	[83]
	A/WS/33 (H1N1) infected MDCK	IC_50_ inhibition: 21.4 ± 2.4 µM	[83]
Myricitrin	A/PR/8/34 (H1N1) infected MDCK	255.6% increase in cell viability at 224.0 µM	[106]

MDCK: Madin-Darby Canine Kidney cells, IC_50_: Half maximal inhibitory concentration, EC_50_: Half maximal effective concentration, HI: Hemagglutination inhibition, NA: Neuraminidase, NAI: Neuraminidase inhibition, RT-PCR: Reverse transcription polymerase chain reaction, PB2: polymerase basic protein 2.

**Table 3:** Selected phenolic compounds detected in *Salicornia* spp. reviewed for their HBV/HCV inhibitory properties. Data are presented as mean values and standard deviations measured by triplicates.

Name	HBV/HCV Inhibition Target	Inhibition Activity	Ref.
Caffeic acid	HCV-1 infected Huh7.5.1 cells	IC_50_ inhibition: 100 ± 20 µM	[132]
	EHMT2	Docking energy: −5.9 kcal/mol	[121]
	HBV-DNA	IC_50_ inhibition: 3.9 ± 1.1 µM	[133]
	HBsAg in vitro assay in infected HepG2.2.2.15 cells	IC_50_ inhibition: 12.7 ± 9.9 µM	[133]
	HBeAg in vitro assay in infected HepG2.2.2.15 cells	IC_50_ inhibition: 109.3 ± 56.0 µM	[133]
Ferulic acid	No data		
Chlorogenic acid	EHMT2	Docking energy: −7.8 kcal/mol	[121]
	STAT3	Docking energy: −7.2 kcal/mol	[121]
	HBV-DNA	IC_50_ inhibition: 1.2 ± 0.4 µM	[133]
	HBsAg in vitro assay in infected HepG2.2.2.15 cells	IC_50_ inhibition: 241.5 ± 198.2 µM	[133]
	HBeAg in vitro assay in infected HepG2.2.2.15 cells	IC_50_ inhibition: >1000 µM	[133]
Apigenin	EC_50_ of HBsAg	EC_50_ inhibition: 26.3 ± 5.6 µM	[134]
	EC_50_ of HBeAg	EC_50_ inhibition: 47.4 ± 3.3 µM	[134]
Kaempferol	HBV-RT	Docking energy: −9.0 kcal/mol	[66]
	HBcAg	Docking energy: −9.1 kcal/mol	[66]
Quercetin	HBV-RT	Docking energy: −8.3 kcal/mol	[66]
	HBcAg	Docking energy: −8.7 kcal/mol	[66]
	NTCP	Docking energy: −5.4 kcal/mol	[66]
	HBsAg in vitro assay in infected HepG2.2.2.15 cells	60% inhibition	[66]
	HBeAg in vitro assay in infected HepG2.2.2.15 cells	62% inhibition	[66]
Isorhamnetin	No data		
Myricetin	Infected fulminant hepatitis mice	66% increased survival rate	[113]
Isoquercitrin	No data		
Myricitrin	HBV-RT	Docking energy: −7.7 kcal/mol	[66]
	HBcAg	Docking energy: −7.1 kcal/mol	[66]
	NTCP	Docking energy: −6.7 kcal/mol	[66]
	HBsAg in vitro assay in infected HepG2.2.2.15 cells	44% inhibition	[66]
	HBeAg in vitro assay in infected HepG2.2.2.15 cells	35% inhibition	[66]

IC_50_: Half maximal inhibitory concentration, EC_50_: Half maximal effective concentration, EHMT2: Euchromatic histone-lysine *N*-methyltransferase 2, HBsAg: Hepatitis B surface antigen, HBeAg: Hepatitis B e antigen, STAT3: Signal Transducer and Activator of Transcription 3, RT: Reverse transcriptase, NTCP: Sodium Taurocholate Cotransporting Polypeptide.

**Table 4:** Selected phenolic compounds detected in *Salicornia* spp. reviewed for their HIV-inhibitory properties. Data are presented as mean values and standard deviations measured by triplicates.

Name	HIV-1 Inhibition Target	Inhibition Activity	Reference
Caffeic acid	IN assay in infected MT-2 cells	IC_50_ inhibition: >278 µM	[149]
	Syncytia formation in Molt-3 cells	93% inhibtion at 255 µM	[150]
	Matrix protein by immunofluorescence analysis	50% inhibtion at 255 µM	[150]
	Capsid protein by immunofluorescence analysis	57% inhibtion at 255 µM	[150]
	RT assay	32% inhibtion at 255 µM	[150]
Ferulic acid	No inhibitory effect	N/A	[151,152]
Chlorogenic acid	IN assay in infected MT-2 cells	IC_50_ inhibition: >142 µM	[149]
	RT by colorimetric enzyme immunoassay	IC_50_ inhibition: 374 µM	[153]
Apigenin	RT by PCR assay	IC_50_: >37.03 µM	[69]
	HIV-1 in infected OM-10.1 cells	IC_50_ inhibition: 12 µM	[148]
Kaempferol	IN integration	IC_50_ inhibition: 64.7 ± 18.1 µM	[146]
	IN cleavage	IC_50_ inhibition: 97.8 ± 9.2 µM	[146]
	RT by PCR assay	IC_50_ inhibition: >34.94 µM	[69]
	PR by fluorescence assay	62.7% inhibition at 174.7 µM	[154]
Quercetin	RT by ELOSA	IC_50_ inhibition: 60 µM	[135]
	PR by oligopeptide cleavage assay	IC_50_ inhibition: >100 µM	[135]
	α-glucosidase inhibition assay	IC_50_ inhibition: >100 µM	[135]
	IN integration	IC_50_ inhibition: 13.6 ± 3.4 µM	[146]
	IN cleavage	IC_50_ inhibition: 23.6 ± 6.6 µM	[146]
	RT by PCR assay	IC_50_ inhibition: <1.65 µM	[69]
	RT by PCR assay	100% inhibition at 6.62 µM	[69]
	RT by ELISA colorimetric enzyme immunoassay	43.41 ± 4.56% inhibition at 661.7 µM	[155]
Isorhamnetin	HIV-1 replication in H9 lymphocyte cells	EC_50_ inhibition: 6.6 µM	[154]
	RT by ELISA colorimetric enzyme immunoassay	56.99 ± 3.91% inhibition at 632.4 µM	[155]
Myricetin	IN integration	IC_50_ inhibition: 2.5 ± 1.0 µM	[146]
	IN cleavage	IC_50_ inhibition: 7.6 ± 0.6 µM	[146]
	RT by PCR assay	IC_50_ inhibition: <1.57 µM	[69]
	RT by PCR assay	100% inhibition at 6.29 µM	[69]
	HIV-1 Blood and lymphocytes infected TZM-bl cells	87% inhibition at 100 µM	[147]
	HIV-1 Blood and lymphocytes infected TZM-bl cells	IC_50_ inhibition: 20.43 µM	[147]
	HIV-1 in infected MT4 cells	EC_50_ inhibition: 230 µM	[156]
	RT assay	IC_50_ inhibition: 7.6 µM	[156]
	RT	Docking energy: −7.0 kcal/mol	[156]
Isoquercitrin	PR by fluorescence assay	64.4% inhibition at 107.7 µM	[154]
Myricitrin	PR by fluorescence assay	50.4% inhibition at 107.7 µM	[154]
	HIV-1 in infected MT4 cells	EC_50_ inhibition: 120 µM	[156]
	RT assay	IC_50_ inhibition: 10.6 µM	[156]
	RT	Docking energy: −5.0 kcal/mol	[156]

IC_50_: Half maximal inhibitory concentration, EC_50_: Half maximal effective concentration, IN: Integrase, RT: Reverse transcriptase, ELOSA: Enzyme-Linked Oligonucleotide Sorbent Assay, PCR: Polymerase Chain Reaction, ELISA: Enzyme-Linked Immunosorbent Assay, PR: Protease.
